# Microarray analysis of the *in vivo *sequence preferences of a minor groove binding drug

**DOI:** 10.1186/1471-2164-9-32

**Published:** 2008-01-23

**Authors:** Todd T Eckdahl, Adam D Brown, Steven N Hart, Kelly J Malloy, Martha Shott, Gloria Yiu, Laura L Mays Hoopes, Laurie J Heyer

**Affiliations:** 1 Biology Department, Missouri Western State University, Saint Joseph, MO, 64507, USA; 2 Mathematics Department, Davidson College, Davidson, NC, 28035, USA; 3 Biology Department, Pomona College, Claremont, CA, 91711, USA; 4 Genome Consortium for Active Teaching, Davidson College, Davidson, NC, 28035, USA

## Abstract

**Background:**

Minor groove binding drugs (MGBDs) interact with DNA in a sequence-specific manner and can cause changes in gene expression at the level of transcription. They serve as valuable models for protein interactions with DNA and form an important class of antitumor, antiviral, antitrypanosomal and antibacterial drugs. There is a need to extend knowledge of the sequence requirements for MGBDs from *in vitro *DNA binding studies to living cells.

**Results:**

Here we describe the use of microarray analysis to discover yeast genes that are affected by treatment with the MGBD berenil, thereby allowing the investigation of its sequence requirements for binding *in vivo*. A novel approach to sequence analysis allowed us to address hypotheses about genes that were directly or indirectly affected by drug binding. The results show that the sequence features of A/T richness and heteropolymeric character discovered by *in vitro *berenil binding studies are found upstream of genes hypothesized to be directly affected by berenil but not upstream of those hypothesized to be indirectly affected or those shown to be unaffected.

**Conclusion:**

The data support the conclusion that effects of berenil on gene expression in yeast cells can be explained by sequence patterns discovered by *in vitro *binding experiments. The results shed light on the sequence and structural rules by which berenil binds to DNA and affects the transcriptional regulation of genes and contribute generally to the development of MGBDs as tools for basic and applied research.

## Background

Improved understanding of the sequence rules by which small molecules bind to DNA and alter patterns of gene expression advances both basic and applied research. In both of these contexts, molecules that bind noncovalently in the DNA minor groove with sequence-selective recognition have drawn considerable attention [1]. Minor groove binding drugs (MGBDs) have served as useful models for protein components of the transcriptional machinery since they can be more experimentally tractable than their macromolecular counterparts. For example, the understanding of the mechanism of action of TATA box binding protein, a general transcription factor required for proper initiation of transcription by RNA polymerase II, has been furthered using the MGBDs distamycin A, Hoechst 33258, and netropsin [2]. The observation that the MGBD berenil affects mitochondrial function and aerobic respiration in yeast suggests that it alters genome-wide patterns of gene expression [3]. A long standing goal in drug development has been the development of agents that can target specific genes in cells, altering patterns of gene expression in a clinically relevant manner. Advances in the general areas of synthetic organic chemistry, molecular biology and biochemistry and specifically in genomics and functional genomics have made the goal of developing more effective drugs tangible. MGBDs have attracted attention because of their demonstrated antitumor, antiviral, antibacterial, and antitrypanosomal activities [4-7]. For example, brostallicin, a derivative of distamycin A, has been shown to be cytotoxic to tumor cells [8] and underwent a phase I clinical investigation involving patients with advanced solid tumors [9]. MGBDs in the category of lexitropsins have been shown to have anticancer properties as well; moderate cytotoxicity in human MCF-7 breast cancer cells was exhibited by analogues of bis-netropsin [10]. Berenil is a member of a family of MGBDs found to be useful in the treatment of infectious diseases caused by *Pneumocystis jiroveci *and trypanosomes [11], diseases of concern in AIDS patients. The design of MGBDs as agents that have more potency but fewer side effects relies on a more thorough understanding of the ways in which MGBDs effect changes at the level of transcription by interacting with promoter DNA and transcription factors of specific genes in the context of chromatin.

MGBDs have cationic charges, narrow molecular cross section, and concave shape, allowing them to fit into the narrow minor groove of DNA [1]. MGBD binding depends on the spine of hydration, van der Waal's interactions with the floor or walls of the minor groove, charge interactions between cationic drug groups and negative electrostatic sequences, the shapes of base pairs, and the width of the minor groove. Because these features vary in a sequence dependent manner, MGBDs exhibit sequence selectivity properties that have been subject of intense study using *in vitro *binding to oligonucleotides and polymeric DNA. MGBDs have been shown to exhibit a preference for short tracts of A/T-rich sequences [12]. The length of DNA protected by MGBDs varies from 4 to 6 bp [13]. The sequence specificity of the MGBD berenil was investigated by measurement of its binding affinity to hairpin oligonucleotides containing all 512 possible 5-mer sequences [14]. The results showed that of 512 sequences studied, all the sequences that were entirely A+T ranked among the fifty best binding sequences, supporting the conclusion that berenil prefers A/T-rich binding sites. Berenil is also apparently able to discriminate among sequences composed only of A+T. The two most optimal binding sequences found in Boger *et al. *were ATATT and AATAT. Binding of berenil to sequences of the form GGGG(A/T)_4_GGGG was studied using electrospray ionization mass spectrometry and it was found to bind sequences in the order ATAT > AATT > AAAA [15]. DNase I footprinting studies revealed the binding of berenil to ATAT, AATT, TAAT, TTAA, and TATA [13]. Rotational viscometry measurements found the sites of highest berenil binding strength to be alternating helical A/T segments [16]. An overarching conclusion from these studies is that berenil prefers to bind to A/T-rich sequences that are heteropolymeric, with A and T alternating on the same strand.

There is a need to extend the study of the sequence requirements for MGBDs to the context of living cells. We chose to contribute to this effort by investigating the effects of the berenil on yeast gene expression, enabling an examination of its *in vivo *sequence binding requirements. Our experimental data and analysis promise to contribute to the body of knowledge of MGBDs with basic and applied research applications.

## Results and Discussion

### Measuring effects of berenil on yeast mRNA levels

The approach we took to finding putative *in vivo *binding sites for berenil was to consider the yeast genome as a bank of DNA sequences that, in the context of chromatin and the environment of the nucleus, have various affinities for the drug. Among these are sequences whose role in the regulation of transcription could be affected by berenil binding. Changes in gene expression at the level of transcription could therefore occur directly through mechanisms such as interfering with transcription factor binding or altering chromatin structure [2,17]. Indirect effects on transcript levels may also occur through gene regulatory networks or general stress response [18]. We sought to use microarray analysis to determine the set of yeast genes that are directly or indirectly affected at the level of transcription by berenil. We then addressed the significant challenge of distinguishing between these two categories during analysis of the upstream regions of affected genes.

### Generation of an affected gene list

Our experimental method was to culture yeast in the presence and absence of berenil, isolate total RNA, and conduct microarray hybridizations to measure changes in steady state transcript levels for all yeast genes. We gathered data suitable for analysis from five experiments, with two whole-genome microarrays in each experiment and two of them conducted as dye swaps of the other three. We analyzed microarray data using MicroArray Genome Imaging and Clustering (MAGIC) Tool [19]. Once MAGIC Tool produced files of foreground and background intensities for all of the microarray experiments, we used Excel to analyze the data. Differences in the performance of the two dyes were accounted for by normalization [20]. A series of filtering criteria were used to ensure that only reliable data were used in the production of an affected gene list. As described in the Methods section, the criteria for inclusion of the data for a given ORF were 1) the sum of the intensities for the two channels had to be greater than the minimum median of that sum for all the experiments, 2) the foreground for at least one channel had to be double the background, 3) data for a feature had to pass the first two filters in at least 5 of the 10 measurements of that feature, 4) data for a feature must pass the first two filters in both Cy3- and Cy5-labelled samples, and 5) the coefficient of variation of log transformed ratios across experiments had to be less than one. The culmination of our analysis was a list of ORFs for which we had reliable microarray data. This resulted in a final list of 52 genes whose mRNA levels decreased and two whose levels increased upon berenil treatment. We find it very interesting that the vast majority of the reliably affected genes were negatively affected; this observation is likely to be relevant to discovery of the mechanism of action of berenil and other MGBDs.

### Real time PCR validation of microarray results

In order to provide validation of the microarray results from an independent method, we performed real time PCR measurements for selected genes. Quantitative reverse transcription real time PCR with SYBR Green reporting was used to generate the data presented in Table 1. The gene TUB1 was used as a standard as described [21] and was also unaffected in our microarray experiments. The genes STF2, HSP78, and SPI1 were found to have lower steady state levels of mRNA according to the microarray data and also had lower levels according to real time PCR. Although the genes appeared in the same order with regard to the magnitude of the effect, the real time data resulted in higher expression ratios, an effect that may be due to signal saturation in the microarray experimental approach.

**Table 1:** Validation of microarray data with real time PCR

**Gene**	**Microarray**	**Real Time PCR**
TUB1	-0.11	0.00 (standard)
STF2	-1.14	-1.81
HSP78	-1.42	-2.60
SPI1	-1.51	-2.74

Expression ratios (log_2 _transformed treated to untreated) of mRNA levels for selected genes inferred from microarray analysis versus real time PCR.

### Direct and indirect effects of berenil

The list of genes whose steady state transcript levels were shown by our microarray analysis to be reliably affected by berenil includes two genes whose levels increased. According to the Saccharomyces Genome Database (SGD), their functions are in phosphate metabolism and processing of 20S pre-RNA [22]. Of the 52 genes whose mRNA levels decrease, 14 are involved in stress response, nine in carbohydrate metabolism, four in electron transport, two in meiosis and mitosis, one in regulation of redox homeostasis, one in regulation of proteolysis, one in salinity response, one in vacuole fusion, one in response to metals, one in phosphate metabolism, and one in DNA repair, according to the SGD. The remaining 16 genes have not had functions assigned to them by SGD. The list of affected genes is likely to include some that are directly affected by berenil. Genes in this category are expected to have upstream transcriptional control regions that include berenil binding sites. Genes whose transcript levels changed by indirect drug effects are also likely to be in the list. Such genes would not be expected to contain berenil binding sites in their upstream control regions. Although we cannot determine which genes may be indirectly affected through gene regulatory networks, we note that there are a number of genes that function in stress response. We chose to test the hypothesis that these 14 genes are indirectly affected by berenil and that the remaining genes are directly affected. Table 2 lists the 40 genes hypothesized to be directly affected while Table 3 lists those hypothesized to be indirectly affected, each with an expression ratio, function, and molecular process, if known.

**Table 2:** Direct hypothesis category of yeast genes shown by microarray analysis to be affected by berenil treatment

**ORF**	**Gene**	**Ratio**	**Function**	**Process**
YBR233W-A	DAD3	-3.45	mitosis	protein binding activity
Q0130	OLI1	-2.75	ATP synthase activity	ATP synthesis coupled proton transport
YDR070C	FMP16	-2.43	unknown	unknown
YEL039C	CYC7	-2.41	electron transport	electron carrier activity
YLR327C	TMA10	-2.35	unknown	unknown
YJL156W-A	YJL156W-A	-2.25	unknown	unknown
YMR105C	PGM2	-2.23	glucose 1-phosphate utilization	phosphoglucomutase activity
YGR248W	SOL4	-2.00	unknown	unknown
YHR087W	YHR087W	-1.95	unknown	unknown
YPR160W	GPH1	-1.76	glycogen catabolism	glycogen phosphorylase activity
YLR178C	TFS1	-1.76	regulation of proteolysis	lipid binding activity
YOR173W	DCS2	-1.72	unknown	unknown
YEL011W	GLC3	-1.66	glycogen metabolism	1,4-alpha-glucan branching enzyme activity
YMR081C	ISF1	-1.64	aerobic respiration	unknown
YER150W	SPI1	-1.51	unknown	unknown
YER067W	YER067W	-1.50	unknown	unknown
YOR031W	CRS5	-1.45	response to metal ion	copper ion binding activity
YIL136W	OM45	-1.43	unknown	unknown
YPL230W	YPL230W	-1.41	unknown	unknown
YOR178C	GAC1	-1.38	meiosis	protein phosphatase type 1 activity
YFR053C	HXK1	-1.35	fructose metabolism	hexokinase activity
YOR120W	GCY1	-1.33	salinity response	aldo-keto reductase activity
YFR017C	YFR017C	-1.32	unknown	unknown
YOR374W	ALD4	-1.31	ethanol metabolism	aldehyde dehydrogenase (NAD+) activity
YJR096W	YJR096W	-1.29	arabinose metabolism	oxidoreductase activity
YFR015C	GSY1	-1.27	glycogen metabolism	glycogen (starch) synthase activity
YDR453C	TSA2	-1.22	regulation of redox homeostasis	thioredoxin peroxidase activity
YHL021C	FMP12	-1.21	unknown	unknown
YKL151C	YKL151C	-1.15	unknown	unknown
YGR008C	STF2	-1.14	ATP synthesis	unknown
YDL130W-A	STF1	-1.09	ATP synthesis	unknown
YCL042W	YCL042W	-1.08	unknown	unknown
YOR385W	YOR385W	-1.07	unknown	unknown
YIL045W	PIG2	-1.07	unknown	protein phosphatase regulator activity
YLR258W	GSY2	-1.06	glycogen metabolism	glycogen (starch) synthase activity
YMR173W	DDR48	-1.04	DNA repair	unknown
YNL015W	PBI2	-1.03	vacuole fusion (non-autophagic)	endopeptidase inhibitor activity
YCL040W	GLK1	-1.02	carbohydrate metabolism	glucokinase activity
YAR071W	PHO11	1.02	phosphate metabolism	acid phosphatase activity
YKL099C	UTP11	1.18	processing of 20S pre-rRNA	snoRNA binding activity

Genes hypothesized to be directly affected by berenil treatment are listed with their expression ratios (log_2 _transformed treated to untreated) and functions, if known.

**Table 3:** Indirect hypothesis category of yeast genes shown by microarray analysis to be affected by berenil treatment

**ORF**	**Gene**	**Ratio**	**Function**	**Process**
YOL052C-A	DDR2	-2.44	response to stress	unknown
YGR088W	CTT1	-2.35	response to stress	catalase activity
YBR072W	HSP26	-2.32	response to stress	chaperone activity
YML100W	TSL1	-2.17	response to stress	enzyme regulator activity
YCR021C	HSP30	-2.17	response to stress	heat shock protein activity
YFL014W	HSP12	-1.93	response to oxidative stress	heat shock protein activity
YMR250W	GAD1	-1.66	response to oxidative stress	glutamate decarboxylase
YMR169C	ALD3	-1.64	response to stress	aldehyde dehydrogenase
YNL160W	YGP1	-1.42	response to stress	unknown
YDR258C	HSP78	-1.42	response to stress	chaperone activity
YDR074W	TPS2	-1.28	response to stress	trehalose phosphatase
YKL026C	GPX1	-1.24	response to oxidative stress	glutathione peroxidase
YLL026W	HSP104	-1.18	response to stress	heat shock protein activity
YLL039C	UBI4	-1.18	response to stress	protein tagging activity

Genes hypothesized to be indirectly affected by berenil treatment are listed with their expression ratios (log_2 _transformed treated to untreated) and functions, if known.

### Sequences found upstream of affected genes

We sought to analyze the upstream regions of the hypothesized direct and indirect categories of genes for the occurrence of sequence features and to consider the results in light of *in vitro *berenil binding studies. In this way, we can address the validity of our categorization of the affected genes and extend knowledge of berenil binding preferences to the environment of cells. As reviewed above, studies conducted *in vitro *have shown that berenil binds 5–6 nucleotide A/T-rich tracts that tend toward heteropolymeric (alternating A and T) character [13-16]. If these binding preferences extend to the cellular context, then the upstream sequences of the 40 yeast genes hypothesized to be directly affected by berenil and listed in Table 2 are expected to contain 5–6 nucleotide sequence elements that are A/T-rich and heteropolymeric. For the 14 genes hypothesized to be indirectly affected and listed in Table 3, the signal of berenil binding sequences should fall to the background level found in the upstream sequences of all yeast genes. Our challenge was to find ways to analyze the sequences in order to uncover any existing sequence patterns. We reasoned that if the effect of berenil on transcription levels is due to binding sites that they would be found upstream of directly affected genes as 5-mer and 6-mer sequences. In order to reduce the background noise, we limited our search to 200 nt upstream of the start site for translation and to the sense strand only. We developed two measures for determining whether the occurrence rate of a given sequence element is unusually high in the upstream regions of genes. The first was the difference between the percentages of the upstream regions of affected and unaffected genes containing a sequence. The second is the ratio of the number of occurrences of a sequence in the affected gene upstream regions to that of the unaffected gene upstream regions.

For use as a control group, we assembled a list of 56 genes that remained reliably unaffected by berenil treatment in the course of our microarray experiments. Sense strand sequences from the 200 bp upstream of these genes, the 40 directly affected genes, and the 14 indirectly affected genes were measured for the occurrence of all possible 5-mer and 6-mer sequences The percentage of genes with an. occurrence of each sequence was determined for each of the three categories and the difference in percentage was calculated between each of the two affected categories and the unaffected category.

Table 4 shows the top ten 5-mer and 6-mer sequences from the direct category according to this criterion. For example, AATAA occurred upstream of 71% of the directly affected genes, but in only 40% of the unaffected ones, for a difference of 31%. The sequences listed occur in an average of 61% of the direct gene upstream regions. All of them occur more frequently upstream of direct category genes than of the unaffected genes; the average occurrence is 23% higher. We also measured the number of occurrences of each sequence in the directly affected, indirectly affected, and unaffected gene upstream regions. The ratio of the number of occurrences in the affected category to that in the unaffected one was calculated and the sequences were ranked according to this ratio, with the top ten sequences listed in Table 5. For example, the sequence ATAAG occurs in 30 times in the 40 affected gene upstream regions, a rate that is 2.3 times higher than in the unaffected gene regions. The sequences listed occur an average of 28 times in the 40 directly affected genes, an average of 2.1 times the occurrence rate found in the 56 unaffected gene regions.

**Table 4:** Difference criterion sequences in direct gene category

**5-mer**	**Directly Affected**	**Difference**	**6-mer**	**Directly Affected**	**Difference**
**aataa**	71%	31%	**tatata**	61%	33%
**ataag**	58	29	**atataa**	58	32
**agaat**	50	25	**aaaaga**	45	23
**aacaa**	55	24	**aaataa**	45	21
aaata	74	23	aaaata	37	20
**atata**	74	23	**tataag**	34	20
**tataa**	74	23	**taataa**	32	19
cataa	42	22	**gaaata**	29	18
**gtaaa**	47	22	aaagaa	47	16
aaaga	66	19	**aataat**	29	16

Sequences found to be most overrepresented in the upstream regions of genes hypothesized to be directly affected by berenil compared to unaffected genes. The percentage of affected genes and difference in percentage between affected and unaffected genes having each sequence is listed. Bolded sequences are shared with Table 5.

**Table 5:** Ratio criterion sequences in direct gene category

**5-mer**	**Directly Affected**	**Ratio**	**6-mer**	**Directly Affected**	**Ratio**
**ataag**	30	2.3	**tataag**	15	2.6
**aacaa**	36	2.1	**atataa**	28	2.6
**aataa**	48	2.1	aatata	19	2.4
**gtaaa**	25	2.1	**gaaata**	12	2.4
taata	33	1.8	**taataa**	15	2.3
acata	25	1.8	**tatata**	35	2.2
**agaat**	24	1.8	**aataat**	14	2.2
**atata**	56	1.7	**aaataa**	22	2.1
**tataa**	42	1.7	ataata	16	2.0
tatat	48	1.5	**aaaaga**	20	2.0

Sequences ranked according to the number of occurrence in upstream regions of genes hypothesized to be directly affected compared to unaffected genes. The number of occurrences in affected genes and ratio of occurrences in affected genes to unaffected genes is listed for each sequence. Bolded sequences are shared with Table 4.

Table 6 shows the top ten sequences according to the difference criterion for the indirect gene category. For example, ACCTC occurs in 50% of the indirect gene regions but only 2% of the unaffected one, for a difference of 48%. The sequences listed occur in an average of 50% of the 14 genes hypothesized to be indirectly affected with an average difference of 35% between the affected and unaffected gene regions. Table 7 shows the results of ranking sequences in the upstream region of genes in the indirect category using the ratio criterion. For example, the sequence AATCT occurs nine times in the indirect sequence regions, a rate that is 3.3 times higher than that found for the unaffected genes. The sequences listed occur an average of 7.8 times in the upstream regions of the 14 indirectly affected gene set, an average of 4.4 times higher than the rate found for the 56 unaffected genes.

**Table 6:** Difference criterion sequences in indirect gene category

**5-mer**	**Indirectly Affected**	**Difference**	**6-mer**	**Indirectly Affected**	**Difference**
acctc	50%	48%	**ctgaaa**	42%	38%
**aatct**	58	42	**taagga**	42	38
ctaat	58	40	atataa	58	33
ctcac	50	37	**aaacaa**	50	32
cttat	50	37	**aaacca**	42	31
gatta	50	37	**aataca**	42	31
**aacaa**	67	36	**tctttc**	42	31
ataca	67	36	ataaat	50	30
aaagc	50	35	acacat	33	30
**acaca**	58	35	ctcacc	33	30

Sequences found to be most overrepresented in the upstream regions of genes hypothesized to be indirectly affected by berenil compared to unaffected genes. The percentage of affected genes and difference in percentage between affected and unaffected genes having each sequence is listed. Bolded sequences are shared with Table 7.

**Table 7:** Ratio criterion sequences in indirect gene category

**5-mer**	**Indirectly Affected**	**Ratio**	**6-mer**	**Indirectly Affected**	**Ratio**
**aatct**	9	3.3	cattct	5	20.0
**aacaa**	16	2.7	**ctgaaa**	5	10.0
caaca	8	2.5	**taagga**	5	10.0
caata	8	2.3	aacaac	5	5.0
aatac	8	2.1	acaaca	5	5.0
tctct	9	2.1	tataag	7	3.5
**acaca**	8	2.0	**aaacca**	5	3.3
tataa	17	2.0	**aataca**	5	3.3
ataag	8	1.8	**tctttc**	5	3.3
gaata	9	1.6	**aaacaa**	9	3.0

Sequences ranked according to the number of occurrence in upstream regions of genes hypothesized to be indirectly affected compared to unaffected genes. The number of occurrences in affected genes and ratio of occurrences in affected genes to unaffected genes is listed for each sequence. Bolded sequences are shared with Table 6.

The difference and ratio criteria for choosing overrepresented sequences yield more similar results for the direct category than for the indirect category. Of the 20 sequences from the direct category listed in Table 4, 15 are found in Table 5, shown in bold. Only nine indirect category sequences are shared between Tables 6 and 7. The extent to which the difference and ratio lists share sequences can be attributed to three causes. First, the two measures are not unrelated. The number of occurrences of a given sequence affects the number of genes with which it is associated, and vice versa. Second, an average of one shared sequence is expected at random. Third, shared sequences may occur because they reflect sequence characteristics of the data set. Since the first two of these causes are not expected to be different for the direct and indirect categories, the increased level of shared sequences for the direct category is telling. It is likely to arise from characteristics of the upstream regions of the direct category genes. In order to investigate these characteristics, we formed sets of unique 5- and 6-mers for the direct and indirect categories that included each shared sequence only once and conducted several types of sequence analysis.

### Sequence analysis

Several observations can be made regarding the sequences presented in Tables 4, 5, 6, 7 that can be used to address our hypotheses about genes that are directly or indirectly affected by berenil and to evaluate the extent to which the rules for drug binding *in vitro *can be extended to the cellular context. These observations relate to the A+T content and the extent of heteropolymeric character found in the sequences listed in Tables 4, 5, 6, 7 and are outlined in the following sections.

#### 1. Overall A/T Richness in Direct Category Sequences

The most obvious characteristic of the 5- and 6-mer sequences found to be overrepresented upstream of the direct category genes is A/T richness. The set of unique 5-mers listed for the direct category in Tables 4 and 5 is 89% A+T while the unique 6-mers listed are 94% A+T. For comparison, the A+T content of the 200 nt upstream regions of 5869 yeast genes averages 65%. The results of a Z-test showed a high level of significance (p < .0001) for the A+T content of both the 5- and 6-mers compared to the set of all yeast genes. The 5- and 6-mer sequences from the indirect category in Tables 6 and 7 average 72% and 73% A+T, respectively. These values are not significantly different from all yeast genes, with p-values of 0.19 and 0.14. These observations support the conclusion that A/T richness is a characteristic of the direct category sequences much more than it is of the indirect category sequences.

#### 2. A/T Richness of Individual Direct Category Sequences

Another observation is that the A+T content levels of individual members of the direct category lists are uniformly high. In Tables 4 and 5, 100% of the 5- and 6-mers are at least 80% A+T. The average value of this measure for 5869 yeast genes upstream regions is only 35% and 19% for 5-mers and 6-mers, respectively. By contrast, of the indirect category sequences of Tables 6 and 7, only 65% of the 17 unique 5-mers and 43% of the 14 unique 6-mers are at least 80% A+T. The high A+T content of individual 5- and 6-mers from the direct category means that none of them contains more than one C or G nucleotide. Interestingly, each time a C or G occurs, it is either at the end of the sequence or it disrupts a 2–5 nt homopolymeric A stretch.

#### 3. High Rate of AT and TA Dinucleotides in Direct Category

The occurrence of heteropolymeric AT and TA dinucleotides is unusually high in the direct category compared to the indirect category. Among the 25 unique 5- and 6-mers in Tables 4 and 5 from the direct category, 52% of the dinucleotides are AT and TA. Based on the number of As and Ts in the sequences, a level of 33% is expected by chance. Of the dinucleotides in the 31 unique indirect category sequences of Tables 6 and 7, 25% are AT and TA, while 23% are expected by the number of As and Ts. For the analogous region upstream of 5869 yeast genes, 18% of dinucleotides are AT or TA, with an expected value of 21% based on As and Ts.

#### 4. Occurrence of Completely Heteropolymeric A/T Sequences

The completely A/T heteropolymeric sequences ATATA, TATAT, ATATAT, and TATATA occur at high rates in the direct category. Of eight possible sequences that are 100% alternating As and Ts that could have occurred in Tables 4 and 5, five appear. Based on A+T content, only 1.1 occurrences would be expected at random. By contrast, none of these sequences occurs in the indirect category lists of Tables 6 and 7. This observation is best explained by lower A+T content; an average of 0.34 occurrences would be expected based on A+T content.

#### 5. Direct Category Sequences Enriched for Heteropolymeric A/T Tracts

There is a high rate of occurrence of 2–6 nt heteropolymeric A/T tracts among the set of unique 5- and 6-mers from the direct gene category. In order to investigate statistical significance of this observation, we compared the rate of occurrence of heteropolymeric A/T tracts for both the direct and indirect category to that found in the 200 nt upstream regions of 5869 yeast genes, and the results are listed in Table 8. Heteropolymeric tracts of 2–5 nt in length occur an average of 7.9 times more often in the unique direct category 5-mers than in the set of 5869 yeast genes and 8.6 times more often in the unique direct category 6-mers. Among the unique indirect category 5- and 6-mers, the heteropolymeric tracts occur at average rates of only 1.3 and 1.9 times higher than in the 5869 yeast genes. We also conducted a one-sided Z-test of the rates of occurrence of the heteropolymeric A/T tracts in both the direct and indirect categories compared to the set of all yeast genes. Although the significance levels are inflated by the lack of independence in overlapping heteropolymeric A/T tracts, this affects both the direct and indirect categories equally, so the resulting p-values can be fairly compared. As shown in Table 8, p-values of less than 0.0001 indicate very high levels of statistical significance for each of the nine direct category comparisons. These results indicate that heteropolymeric A/T tracts of 2–6 nt occur at a higher rate in the direct category sequences compared to yeast genes in general. Enrichment of heteropolymeric A/T tracts in the indirect category compared to the 5869 yeast genes is far less significant, and the significance levels decrease as the tract length increases.

**Table 8:** Analysis of A/T heteropolymeric sequence occurrences

**A/T Heteropolymeric Sequences**		**5-mers**		**6-mers**	
		
		**Direct**	**Indirect**	**Direct**	**Indirect**
**AT, TA**	observed	0.500	0.294	0.533	0.214
	yeast	0.180	0.180	0.180	0.180
	p-value	**< .0001**	0.007	**< .0001**	0.228
					
**ATA, TAT**	observed	0.359	0.157	0.396	0.125
	yeast	0.065	0.065	0.065	0.065
	p-value	**< .0001**	0.004	**< .0001**	0.033
					
**ATAT, TATA**	observed	0.192	0.029	0.222	0.071
	yeast	0.024	0.024	0.024	0.024
	p-value	**< .0001**	0.418	**< .0001**	0.022
					
**ATATA, TATAT**	observed	0.154	0.000	0.167	0.036
	yeast	0.010	0.010	0.010	0.010
	p-value	**< .0001**	0.662	**< .0001**	0.090
					
**ATATAT, TATATA**	observed	-	-	0.083	0.000
	yeast	-	-	0.010	0.010
	p-value	-	-	**< .0001**	0.579

The rate of occurrence (observed) of 2–6 nt A/T heteropolymeric sequences in the unique 5-mer and 6-mer sequences listed in Tables 4-7 for the direct and indirect gene categories was compared to the rate of occurrence (yeast) in the 200 nt upstream region of 5869 yeast genes using a 1-sided Z-test. Highly significant p-values are shown in bold.

#### 6. Basis for Heteropolymeric A/T Tracts

Clearly, the occurrence of heteropolymeric A/T tracts is higher in the upstream regions of the direct category genes than in the corresponding regions of the indirect category genes or of yeast genes in general. But to what extent can this be attributed to the A/T richness of these regions or to the high rate of occurrence of A/T tracts of any type? We sought to address these questions by conducting an analysis of the rate of occurrence of heteropolymeric sequences compared to the rate expected by either A+T content or the occurrence of 100% A/T tracts. We first tabulated the number of occurrences of 3–6 nt A/T heteropolymeric tracts in each of the eight lists of Tables 4, 5, 6, 7. We then used two different means to establish an expected number of occurrences of these sequences. One was simply A+T content, with the consequence that higher content results in more expected occurrences of the A/T heteropolymeric tracts. The other involved using the number of 100% A/T tracts that occurs in a given list to determine the expected number of A/T heteropolymeric tracts. For 3 nt A/T tracts, two out of eight are expected at random to be ATA or TAT. For 4 nt tracts, the expected rate is two of 16, for 5 nt two of 32, and for 6 nt two of 64. For the direct sequences listed in Table 4, A/T heteropolymeric tracts of all sizes occur at rates greater than expected by A+T content, with an average ratio of observed to expected of 3.3. The same is true for the direct category sequences in Table 5, with an average ratio of 4.2. However, A/T heteropolymeric tracts in the sequences from the indirect lists in Tables 6 and 7 occur near the expected frequencies, with ratios of observed to expected of 1.8 and 0.6, respectively. Using the expected values from the occurrence of 100% A+T tracts, the sequences of Tables 4 and 5 still display unusually high occurrences of A/T heteropolymeric tracts, with ratios of observed to expected of 3.0 and 3.6, respectively. However, the indirect sequence category has the expected sequence properties since the ratio of observed to expected for Table 6 is 1.2 and the ratio for Table 7 is 0.8. We also conducted a Chi-squared analysis of the occurrence of 3–6 nt A/T heteropolymeric sequences in each of the lists from Tables 4, 5, 6, 7. Strikingly, for the unique direct category sequences there is high degree of statistical significance for the occurrence of A/T heteropolymeric tracts based on A+T content in every one of the seven combinations of tract length and 5-mer versus 6-mer (p-values range from < .0001 to .018). All seven direct category combinations also yielded a high degree of significance when the expected values were based on A/T tracts (p-values from < .0001 to .013). Equally striking is the result that for the unique indirect category sequences, none of the fourteen analyses showed statistical significance (p-values from .13 to .89), indicating that A/T heteropolymeric tracts occur at expected frequencies.

## Conclusion

The results of our microarray experiments and associated sequence analysis provide insight into the sequence patterns required for binding of the minor groove binder berenil in the environment of yeast cells. They support the conclusion that the upstream regions of the genes hypothesized to be directly affected by berenil contain sequence features that are in good accord with those discovered by *in vitro *berenil binding studies. This was established by observation of sequence characteristics of the upstream regions of the direct category genes: high A+T content, A/T richness of the most frequently found 5- and 6-mers, and a high rate of occurrence of 2–6 nt heteropolymeric A/T sequences. By contrast, these sequence features were not apparent in the upstream regions of the genes hypothesized to be indirectly affected by berenil or by those shown to be unaffected by the treatment.

Our hypotheses about which genes were directly affected by berenil and which were indirectly affected were based solely on information about their functions. However, each of the ways that we analyzed the sequences found upstream of the genes supported the conclusion that the direct category upstream regions contained sequence characteristics found by *in vitro *binding studies while the indirect category regions did not. These hypotheses would benefit from further experimentation on individual genes and on the mechanism by which direct and indirect effects are manifested.

Our observation that 52 of the 54 affected yeast genes were negatively affected by berenil may have important implications for the mechanism of action of the drug, directing us to several possible mechanisms of drug binding that can be expressed as testable hypotheses. One hypothesis is that the drug interferes with the binding of transcription factors to DNA upstream of affected genes. This hypothesis is supported by several studies. For example, MGBDs have been shown to compete with the transcription factor NF-Y for binding to the DNA minor groove [23] and to both prevent and disrupt binding of TBP to it [24]. Of the 25 unique sequences listed in Tables 4 and 5 from the upstream regions of the direct category genes, five are exact matches to the TBP consensus binding site of TATAWAW [25]. There are also three matches to the TBP consensus among the indirect category sequences of Tables 6 and 7. TBP was found to bind the TATA box sequence TATATAAA from the yeast CYC1 gene [26]. Seven exact matches to this sequence are found of all four direct category lists while only two are found in the indirect lists. HAP1 is a zinc finger transcription factor of the Zn(2)-Cys(6) binuclear cluster domain type that is known to make minor groove contact with the sequence GCTAATAGCGATAATAGCGAGGG [27]. This sequence includes two exact matches to the unique direct sequences listed in Tables 4 and 5 and only one match to the unique indirect sequences in Tables 6 and 7. It is also found in the upstream region of CYC7, a gene whose expression level was found to be lowered by berenil in our study.

A second hypothesis is that berenil is able to affect the initiation of transcription by altering the conformation of DNA in promoter sequences. Evidence points to the ability of MGBDs to bind to the narrowed minor groove of A/T tracts spaced at a periodicity that produces intrinsic DNA curvature; uncurving of naked DNA by MGBDs has also been demonstrated [28]. In order to make predictions of DNA curvature for the upstream regions of our affected genes, we used bend.it ^® ^[29] to calculate predicted curvature in 500 bp regions upstream of the 54 genes shown by our study to be affected by berenil [30]. Regions of strongly predicted curvature were notably absent; the maximum was 2.4 degrees/helical turn and the average value was 2.2 degrees/helical turn whereas DNA sequences shown experimentally to demonstrate curvature give values between 10 and 20 degrees/helical turn [30]. Although these observations do not support the hypothesis that berenil affects the regulation of transcription by uncurving DNA, the possibility remains that it introduces DNA curvature.

A third hypothesis is that MGBDs cause changes in chromatin structure indirectly through effects on DNA curvature. Curved DNA has been shown to position nucleosomes in reconstitution studies, to affect the stability of nucleosomes, and to disrupt nucleosomes assembled onto curved DNA [17,31]. Perhaps berenil negatively influences transcription by preventing upstream sequences from interacting with nucleosomes and positively affects transcription by facilitating the interaction of upstream sequences with nucleosomes. A testable model is that a nucleosome is positioned at upstream sequences in a way that allows access at adjacent downstream linker regions for transcription factors. For a gene that is expressed, the upstream sequence would position the nucleosome. Binding of berenil could alter the DNA curvature in a way that would prevent the nucleosome positioning and reduce access by the transcription factor. Conversely, a gene that is not as active in transcription may have a sequence that, when bound by berenil, adopts a conformation that attracts a positioned nucleosome and makes a transcription factor binding site available, up-regulating transcription. Perhaps the former of these two mechanisms occurs more readily, since the vast majority of the affected genes in our study were negatively affected by berenil.

Clearly, much more can be learned about the mechanism by which berenil and other MGBDs affect gene expression at the level of transcription. However, the results reported here provide an extension from *in vitro *binding studies to experiments involving cells. Extension of this work promises to contribute to the further development of MGBDs as tools for basic research and drugs with important clinical applications.

## Methods

### Yeast culture and RNA isolation

Yeast were inoculated at a level of A_660 _= 0.2 and cultured at 30C for four hours in the presence and absence of 10 μM berenil. The incubation time was chosen in order to expose the yeast to berenil for two generations and the concentration was that required for a measurable effect of berenil on yeast colony size (data not shown). However, no effect on growth as measurable by A_660 _was observed over the course of the treatment. Total RNA was isolated from the two yeast cultures using the Ambion Ribopure Yeast kit and tested for quality with both UV absorbance and agarose gel electrophoresis.

### Microarray hybridizations

Using the Genisphere Array 900 kit, we prepared cDNA from mRNA for the two RNA populations and conducted a two step hybridization involving Cy3 and Cy5 fluorophores to a DNA microarray spotted with 70 mer oligos that represent the known reading frames in the yeast genome. Hybridized microarrays were then scanned using the GCAT scanner at Davidson College to generate images that could be subjected to microarray analysis. We used the Cy3 and Cy5 dyes in opposite ways in replicate experiments (so-called dye swapping). We gathered data suitable for analysis from a total of five different microarray experiments, with two of them conducted as dye swaps of the other three. Moreover, each experiment included two copies of the oligo set for all of the yeast open reading frames.

### Microarray data analysis

In order for the microarray data for a given gene to be used, the sum of the intensities for the two channels had to be greater than the minimum median of that sum for all the experiments and the foreground for at least one channel had to be double the background. In addition, data passing these criteria had to come from at least 5 of the 10 experiments and be represented in Cy3- and Cy5-labelled samples. Finally, the coefficient of variation of log_2 _transformed ratios across experiments had to be less than one. The culmination of our analysis was a list of ORFs for which we had reliable microarray data. We chose to limit the list to those for which the effect of drug treatment was two-fold or greater.

### Real time PCR

Quantitative reverse transcription PCR was performed using primer sets designed to amplify a region of about 100 bp near the 3' end of the mRNA. An ABI Prism 7000 followed the reactions run in 96 well plates using the standard curve method. Three replicates were performed for each concentration for the standard curve, and 4 replicates for each gene. Reactions were set up using the ABI RT kit and SYBR green reagent mixture. TUB1 served as the internal standard gene [21]. After data were collected, the samples were subjected to thermal denaturation to verify that single species had been synthesized.

### Sequence analysis

We assembled a list of 54 genes that were shown to be affected by berenil treatment in our microarray experiments. These were subdivided into 40 genes hypothesized to be directly affected (Table 2) and 14 genes hypothesized to be indirectly affected (Table 3). We also compiled a list of 56 genes that were reliably unaffected by berenil treatment. For each gene, we measured the occurrence of 5-mer and 6-mer sequences in the 200 nt region upstream of the start site for translation, using the sense strand only The percentage of affected genes that had an occurrence for a sequence was compared to the percentage of control sequences in which the sequence occurred. The 5-mers whose percentage of affected genes was at least two standard deviations above the mean and the 6-mers whose percentage of affected genes was at least four standard deviations above the mean were ranked according to the difference between the percentage for affected and control genes, and the top ten for the direct category are listed in Table 4 while those for the indirect category are listed in Table 6. The number of occurrences of 5-mers and 6-mers was determined for the upstream 200 bp sequences of the 40 direct category genes, the 14 indirect category genes, and the 56 unaffected control genes. The ratio of the number of occurrences of affected versus unaffected was calculated, correcting for the number of genes in each set. The 5-mers whose occurrence was at least two standard deviations above the mean and 6-mers whose occurrence was at least four standard deviations above the mean were ranked according to the ratio of occurrences in affected genes to occurrences in unaffected genes. The top ten sequences are listed in Table 5 for the direct category and Table 7 for the indirect category.

### Microarray Data Availability

All the microarray data generated for this report are available on the Genome Consortium for Active Teaching (GCAT) website [32].

## Authors' contributions

TTE conceived of the study, coordinated the microarray experiments, participated in the microarray data analysis and the sequence analysis, and was primarily responsible for writing the manuscript. ADB performed the microarray experiments and participated in the microarray data analysis. SNH participated in the microarray experiments, the microarray data analysis and the sequence analysis. KJM participated in the microarray experiments and the microarray data analysis. MS participated in the microarray data analysis and the sequence analysis. GY performed the real time PCR experiments. LLMH coordinated the real time PCR experiments and real time PCR data analysis. LJH coordinated the microarray data analysis and the sequence analysis and contributed to the writing of the manuscript. All authors read and approved the final manuscript.

## References

[B1] Neidle S (2001). DNA minor-groove recognition by small molecules. Nat Prod Rep.

[B2] Chiang SY, Welch J, Rauscher FJ, Beerman TA (1994). Effects of minor groove binding drugs on the interaction of TATA box binding protein and TFIIA with DNA. Biochemistry.

[B3] Ferguson LR, Sundberg RJ (1991). Petite mutagenesis in Saccharomyces cerevisiae by a series of bis-cationic trypanocidal drugs. Antimicrob Agents Chemother.

[B4] Baraldi PG, Bovero A, Fruttarolo F, Preti D, Tabrizi MA, Pavani MG, Romagnoli R (2004). DNA minor groove binders as potential antitumor and antimicrobial agents. Med Res Rev.

[B5] Neamati N, Mazumder A, Sunder S, Owen JM, Tandon M, Lown JW, Pommier Y (1998). Highly potent synthetic polyamides, bisdistamycins, and lexitropsins as inhibitors of human immunodeficiency virus type 1 integrase. Mol Pharmacol.

[B6] Cushion MT, Walzer PD, Collins MS, Rebholz S, Vanden Eynde JJ, Mayence A, Huang TL (2004). Highly active anti-Pneumocystis carinii compounds in a library of novel piperazine-linked bisbenzamidines and related compounds. Antimicrob Agents Chemother.

[B7] Turner PR, Denny WA (2000). The genome as a drug target: sequence specific minor groove binding ligands. Curr Drug Targets.

[B8] Beria I, Baraldi PG, Cozzi P, Caldarelli M, Geroni C, Marchini S, Mongelli N, Romagnoli R (2004). Cytotoxic alpha-halogenoacrylic derivatives of distamycin A and congeners. J Med Chem.

[B9] Lockhart AC, Howard M, Hande KR, Roth BJ, Berlin JD, Vreeland F, Campbell A, Fontana E, Fiorentini F, Fowst C, Paty VA, Lankford O, Rothenberg ML (2004). A phase I dose-escalation and pharmacokinetic study of brostallicin (PNU-166196A), a novel DNA minor groove binder, in adult patients with advanced solid tumors. Clin Cancer Res.

[B10] Pućkowska A, Bielawski K, Bielawska A, Midura-Nowaczek K (2004). Aromatic analogues of DNA minor groove binders – synthesis and biological evaluation. Eur J Med Chem.

[B11] Agbe SA, Yielding KL (1993). Effect of verapamil on antitrypanosomal activity of drugs in mice. Acta Trop.

[B12] Lauria A, Montalbano A, Barraja P, Dattolo G, Almerico AM (2007). DNA minor groove binders: an overview on molecular modelling and QSAR approaches. Curr Med Chem.

[B13] Abu-Daya A, Brown PM, Fox KR (1995). DNA sequence preferences of several AT-selective minor groove binding ligands. Nucleic Acids Res.

[B14] Boger DL, Fink BE, Brunette SR, Tse WC, Hedrick MP (2001). A simple, high-resolution method for establishing DNA binding affinity and sequence selectivity. J Am Chem Soc.

[B15] Rosu F, Gabelica V, Houssier C, De Pauw E (2002). Determination of affinity, stoichiometry and sequence selectivity of minor groove binder complexes with double-stranded oligodeoxynucleotides by electrospray ionization mass spectrometry. Nucleic Acids Res.

[B16] Reinert KE (1999). DNA multimode interaction with berenil and pentamidine; double helix stiffening, unbending and bending. J Biomol Struct Dyn.

[B17] Fitzgerald DJ, Anderson JN (1999). Selective nucleosome disruption by drugs that bind in the minor groove of DNA. J Biol Chem.

[B18] Yang YL, Liao JC (2004). Network component analysis of Saccharamyces cerevisiae stress response. Conf Proc IEEE Eng Med Biol Soc.

[B19] Heyer LJ, Heyer LJ, Moskowitz DZ, Abele JA, Karnik P, Choi D, Campbell AM, Oldham EE, Akin BK (2005). MAGIC Tool: integrated microarray data analysis. Bioinformatics.

[B20] Quackenbush J (2002). Microarray data normalization and transformation. Nature Genetics.

[B21] Lesur, Campbell JL (2002). The transcriptome of prematurely aging yeast cells is similar to that of telomerase-deficient cells. Mol Biol Cell.

[B22] Saccharomyces Genome Database. http://www.yeastgenome.org/.

[B23] Ronchi A, Bellorini M, Mongelli N, Mantovani R (1995). CCAAT-box binding protein NF-Y (CBF, CP1) recognizes the minor groove and distorts DNA. Nucl Acids Res.

[B24] Chiang SY, Welch JJ, Rauscher FJ, Beerman TA (1996). Effect of DNA-binding drugs on early growth response factor-1 and TATA box-binding protein complex formation with the herpes simplex virus latency promoter. J Biol Chem.

[B25] Bareket-Samish A, Cohen I, Haran TE (2000). Signals for TBP/TATA box recognition. J Mol Biol.

[B26] Kim JL, Burley SK (1994). 1.9 A resolution refined structure of TBP recognizing the minor groove of TATAAAAG. Nat Struct Biol.

[B27] King DA, Zhang L, Guarente L, Marmorstein R (1999). Structure of a HAP1-DNA complex reveals dramatically asymmetric DNA binding by a homodimeric protein. Nat Struct Biol.

[B28] Albert FG, Eckdahl TT, Fitzgerald DJ, Anderson JN (1999). Heterogeneity in the actions of drugs that bind in the DNA minor groove. Biochemistry.

[B29] bend.it Server. http://hydra.icgeb.trieste.it/~kristian/dna/bend_it.html.

[B30] Vlahovicek K, Kaján L, Pongor S (2003). DNA analysis servers: plot.it, bend.it, model.it and IS. Nucl Acids Res.

[B31] Anselmi C, Bocchinfuso G, De Santis P, Savino M, Scipioni A (1999). Dual role of DNA intrinsic curvature and flexibility in determining nucleosome stability. J Mol Biol.

[B32] Genome Consortium for Active Teaching (GCAT). http://gcat.davidson.edu/berenil/datafiles.html.

